# A bibliometric review on the Water Framework Directive twenty years after its birth

**DOI:** 10.1007/s13280-023-01918-0

**Published:** 2023-09-08

**Authors:** Diego Copetti, Stefania Erba

**Affiliations:** National Research Council of Italy - Water Research Institute (CNR- IRSA), Via del Mulino 19, 20861 Brugherio, Monza-Brianza Italy

**Keywords:** Bibliometric-review analysis, Ecological status, Water Framework Directive, WFD impact, WFD implementation, WFD objectives

## Abstract

The European Water Framework Directive (WFD) is one of the most studied environmental legislations and recently turned twenty. The paper deals with a literature search and analysis of 4120 references related to this Directive. After a period of strong increase in article production (2002–2012) WFD scientific productivity is currently still high (~ 260 papers year^−1^), suggesting a persistent interest of the scientific community on this issue. Most research supporting the WFD was on water sciences, but contributions were also from governance and socio-economic disciplines. Studies on biological quality elements and rivers were prominent. The WFD implementation has seen a strong participation of scientists from all EU countries, and partially also from outside-EU nations. To improve the EU water policy and management, the paper suggests a greater interconnection between WFD and other EU Directives and indicates some emerging environmental issues to which the Directive should address.

**Supplementary Information:**

The online version contains supplementary material available at 10.1007/s13280-023-01918-0.

## Introduction

The Water Framework Directive—WFD (EC 2000) is the milestone of the European Union (EU) legislation for water protection. Adopted in 2000, it aims: “to establish a framework for the protection of inland surface waters, transitional waters, coastal waters and groundwater” as stated in WFD article 1, which also sets out the broad and holistic vision of the Directive. Such vision, at the time of the WFD promulgation, was not fully matched by scientific knowledge. Many key issues were introduced including the concepts of reference conditions (i.e., least disturbed environments) and biological quality elements (i.e., phytoplankton, macrophytes, phytobenthos, macroinvertebrates, and fish) which are at the base of the ecological status definition (Feio et al. 2021). However, all these concepts were defined using a relatively subjective language and needed a strong scientific effort, to be turned into a set of principles that could be applied in regulatory contexts, on a national scale.

WFD implementation is a quite complex process requiring a multi-level approach to be carried out at the level of member state (MS). Management actions must be planned at the scale of the catchment, but to be effective they should be based on analysis at smaller spatial scales (e.g., sub-basin, reach, site). To attain the main WFD objective (i.e., the achievement of a good ecological status for all EU water bodies), a River Basin Management Plan (RBMP) at the river basin district level, including a program of measures, must be produced, and periodically updated. Measures within the RBMPs can be explicitly related to other EU Directives (Carvalho et al. 2019).

Such an innovative approach forced MSs to adapt their assessment and management systems to comply with the WFD requirements. Virtually, all MSs have undertook an intense effort to integrate scientific and technical issues with political, economic, and water governance questions. Economic instruments, such as water pricing and the “the polluter-pays” principle, were introduced as part of WFD “basic measures” (Balana et al. 2011; EC 2000).

The WFD workplan was chronologically strictly defined (Fig. S1 of the supplementary information). The first important deadline coincided with the end of the first RBMP cycle and was set for 2015. Maximum derogation was allowed until 2027, with an intermediate cutoff date in 2021 at the end of the second RBMP cycle (EEA 2012). The final 2027 deadline coincides with the conclusion of the third RBMP cycle and represents the last chance for MSs to introduce adequate measures to meet the WFD quality targets, for groundwater and surface waterbodies (Moss et al. 2020).

Due to its articulated approach to the management of aquatic ecosystems and its ambitious objectives, the WFD has stimulated the scientific community in different ways. In particular, the need to share ecological status targets among MSs, engaged the scientific community in the development of a Common Strategy for WFD Implementation (CIS), since the very beginning of the WFD life (EC 2001). In this context, various guidance documents (i.e., 37 at present) and technical reports have been produced to assist the process of the WFD implementation. These documents were prepared within a rigorous scientific frame and were followed by the publication of research papers (e.g., Buffagni et al. 2007; Erba et al. 2009; Poikane et al. 2014; Phillips et al. 2019). Water scientists and researchers from very different research fields have been involved, including scholars from political, legal, economical, and sociological disciplines (Boeuf and Fritsch 2016). To support this challenge both EU and its MSs have funded several research projects (Hering et al. 2010).

The large bulk of information produced within these activities has made the WFD a reference point even outside the EU borders (e.g., Zick et al. 2006; Zhou et al. 2014), its impact on science and academia is well described by a sentence reported in Moss (2008) in which it is argued that, since its promulgation “It has become almost mandatory to refer to the Directive in any paper concerned with applied aspects of aquatic ecology in Europe.” This influence is documented by the large number of scientific documents produced since the WFD promulgation (e.g., Boeuf and Fritsch 2016).

After 20 years from its birth, it is therefore of interest to draw a picture of the evolution of the scientific production on this EU Directive. Accordingly, we conducted a systematic literature search and analyzed the extracted data through bibliometric review methods. Based on authors' knowledge, this is the first time that such an analysis has been carried out. The state of the art emerging from this analysis is interpreted and discussed critically to outline perspectives and new directions for water management and WFD implementation.

## Data and methods

### Literature search

On June 30, 2021 we carried out a search on the Web of Science™ (WoS) core collection database (i.e., All editions) using two keywords: “water framework directive” or “wfd.” We selected the query term Topic (TS) which includes the following research fields: Title, Abstract, Author Keywords, and Keywords Plus (i.e., keywords assigned by WoS). Research was limited to scientific articles written in English. Therefore, the final query was as follows:

$$\text{TS}=(\text{``Water framework directive''}\;\text{or}\;\text{``WFD''}))\;\text{AND LANGUAGE}:\; (\text{English})\;\text{AND DOCUMENT TYPES}:\; (\text{Article})$$

The entire dataset was exported in a series of text files subsequently merged to form a single data collection. Every record contained 67 fields, including: Author Full Name, Article Title, Source Title, Author Keywords, Abstract, and Publication Year.

### Data processing

The whole dataset (hereafter referred as WFD-References Dataset) was imported in a table (WFD-References Table) of a Microsoft^®^ Access ™ database (WFD-References Database) developed by the authors. The authors then implemented a visual interface (Fig. S2)—WFD-References Check—to facilitate the analysis of the imported records. Firstly the Publication Year field was checked, to discard articles published after December 31, 2020. Then Article Title, Author Keywords, and Abstract fields were carefully examined, to eliminate the references that formally met our query criteria, but that did not deal with the Water Framework Directive, because reporting, at least in one of the WoS searched field, the acronym WFD was used to refer to other topics (e.g., Worst-Fit-Decreasing, Water Film Depth, Weighted Feature Distance function). Bibliometric mappings were carried out using such selected references. The WFD-Reference Check visual interface was then used to extract relevant information from the fields Article Title, Author Keywords, and Abstract to be inserted in the WFD-References Table through a series of YES/NO fields (Fig. S2). This information was used to perform a review analysis. Specific comments to each paper were added using a dedicated text field (i.e., Notes, in Fig. S2).

The R software (https://www.r-project.org/) was employed to perform linear regression analysis (Ordinary Least Squares, OLS). QQ plots were used to visually check the distribution of the standardized residuals. The following diagnostic tests (“lmtest” library) were then carried out on the residuals: t Student (zero mean condition), Shapiro–Wilk (normal distribution), Breusch–Pagan (homoscedasticity), Durbin–Watson (serial independence), adopting a 0.05 *p* value threshold of acceptance (Thode 2002).

In this paper, we define *N*_p_ as the number of published papers per year and *N*_c_ the normalized citations (Bezak et al. 2021). *N*_c_ is calculated for each publication as the ratio between the “total number of citations” and the “number of years from the year when the study was published.” *N*_c_ was introduced as an index of the average citation impact of a scientific article over the entire period of study, to obtain a citation estimate not affected by the age of the paper (Bezak et al. 2021).

Some of the summary statistics used (i.e., research areas) refer to InCites 2.0, built on a single dataset source from the Web of Science™ Core Collection, aggregated by the Research Analytics Integrated Metrics System, and optimized in the InCites Dataset.

### Bibliometric mapping

Bibliometric mapping was carried out using the VOSViewer software (https://www.vosviewer.com/, version 1.6.18), which creates bibliometric maps and extracts relevant bibliometric information (van Eck and Waltman 2010, 2014). Input data can be supplied to the software using different formats, including the text format provided by WoS, used in this paper. In VOSViewer, maps can be represented in three different ways: network, overlay, and density visualization. Here, we describe only the network visualization, adopted in this study. Maps represent bibliometric items (i.e., terms, keywords, scientific publications, scientific journals, research organizations, researchers, or countries) visualized as circles or frames and connected by links of different nature (i.e., co-occurrence, citation, co-authorship, co-citation, or bibliographic coupling) to form a bibliometric network. The item positions are established through specific algorithms defining the relationships between items (van Eck and Waltman 2010).

In a bibliometric map, items are described quantitatively by numerical weight attributes that can be both standard (e.g., number of links with other items) or specific (e.g., the occurrence of a term in co-occurrence maps or the number of documents in citation maps). Items with higher weights are more prominent (towards the foreground) than items with lower weights (towards the background). The size of an item (as well as the font of its label) is also scaled based on the weight attribute (van Eck and Waltman 2010, 2014). Items are grouped in non-overlapping clusters (i.e., an item can belong to only one cluster) using the clustering algorithms described in van Eck et al. (2010) and Waltman et al. (2010).

In this paper, we will present only citation (of scientific journals, countries, and institutions) and co-occurrence (of author keywords) maps. A Citation map uses links between two items where one item (e.g., A) cites the other (e.g., B), without distinguishing between the directions of the link (i.e., AB = BA). In a co-occurrence map, instead, terms that co-occur frequently in a specific text are located close to each other, while terms that have a low co-occurrence frequency are located further away one from another (Rizzi et al. 2014). A thesaurus file has been used to perform an author keywords data cleaning before carrying out the author keywords co-occurrence map. We eliminated all keywords containing “water framework directive” and “wfd” as both terms were used in the WoS core collection database search. The occurrences of “water framework directive” (1379) were one order of magnitude higher than those of the second most frequent keyword and would have masked the visualization of the other terms. A thesaurus file has also been used in the country citation map to amalgamate “England,” “Scotland,” “Northern Ireland,” and “Wales” in the single word “UK.”

To tailor a map, the VOSviewer software allows to lay out specific parameter settings and limitations (van Eck and Waltman 2022). To include only relevant items, we imposed that each item produced at least five scientific articles in citation maps and that a keyword occurred not less than five times in co-occurrence maps. To avoid the presence of unconnected items, the minimum number of links for each item was set to one (i.e., each item must be linked at least with another) in both kind of maps. All the maps were thus produced using a subset of the total items. To optimize the number of clusters (i.e., in a range of 3 to 4) and to facilitate data interpretation, the minimum cluster size (number of items in a cluster) was set up between 10 and 20% of the number of items contained in the subset and the clustering resolution was fixed at 0.9 in all maps (van Eck and Waltman 2022).

### Review analysis

Based on the careful examination of the fields: Article Title, Author Keywords, and Abstract we attributed (using the WFD-References Check visual interface) three main issues (i.e., Water Categories, Disciplines, and Connections) and three further levels of classification to all the references. The adopted classification scheme is reported in Fig. S3, while features of the three classification levels are described in Table S1. To set up the Level 1 of classification (Fig. S3) different specifications were provided for each of the three issues. Eight choices were identified for the Water Categories. The first five refer to the WFD article 2 (i.e., River, Lake, Groundwaters, Transitional, and Coastal Waters) while the sixth Heavily Modified/Artificial (HM/A) refer to the WFD articles 8–9. The last two choices, Water Basins and Others, were defined by the authors to include general categories not dealing with those provided by the WFD. Five options were proposed to classify an article according to Disciplines: Water Sciences, Governance, Socio-economy, Legislation, and Others (Table S1). References have been finally classified for Connections if they quote Other EU Directives and/or if they dealt with the application of the WFD in Extra-EU countries. Level 2 details only Water Sciences which were further classified in 11 different groups (Land Use, Water Quantity, Water Quality, Habitat Indicators, Trophy Indicators, Biological Indicators, Chemical Indicators, Wastewater Treatment, Ecosystem Services, Restoration, and Others).

Level 3 provides specifications for two of the items particularly relevant in the implementation of the WFD: Biological and Chemical Indicators. This level included 9 classes (Table S1), 6 for Biological Indicators (Phytoplankton, Phytobenthos, Macrophytes, Macroinvertebrates, Fish, and Others), and 3 for Chemical Indicators (Inorganic pollutants, Organic pollutants, and Ecotoxicology).

## Results

### Descriptive statistics and trends

We retrieved from the WoS core collection database 4566 bibliometric records, 446 of them (9.8%) were discarded because referred to scientific articles published after 2020 (142; 3.1%) or because not dealing with the Water Framework Directive (304; 6.7%). Overall, 4120 bibliometric records (WFD-References Dataset) were thus examined (Table 1). All these papers were classified by WoS (see “Data processing”–“Data and methods” section) at least in one research area. The main research areas assigned by WoS were: Environmental Sciences & Ecology (42%), Engineering (12%), Marine & Freshwater Biology (12%), Biodiversity & Conservation (7%), and Water Resources (4%), the remaining 23% of the papers was classified in other 44 minor areas (Table S2). Percentages were calculated using only the first research area, even when more than one area was assigned. The first scientific article in the dataset was published in 1998 (Pollard and Huxham 1998), 2 years before the Directive promulgation. The main bibliometric indicators of the WFD-References Dataset are reported in Table 1.

**Table 1:** Main bibliometric indicators of the WFD-References Dataset

Index	Value
Publication timespan	1998–2020
Papers	4120
Journals	591
Authors	12 865
Institutions	3475
Countries	92
Citations	98 556
References	121 245
Author keywords	8893
Paper average age	7.6
Paper average citation per paper	23.9
Average co-authors per paper	3.1

**Fig. 1:**
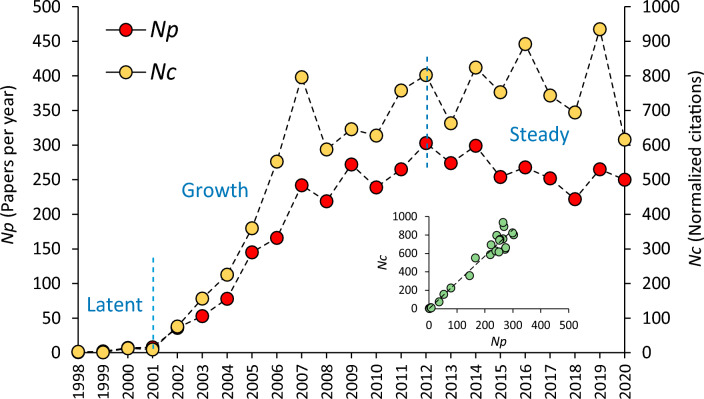
Main panel: trend of the number of papers published per year (*N*_p_) and normalized citations (*N*_c_) in the period 1998–2020, vertical dashed lines separate the three main phases of the trend: Latent, Growth, and Steady phase. Lower right panel: relationship between *N*_p_ and *N*_c_

The year with the lowest and the highest number of published papers per year (*N*_p_) was 1998 (1) and 2012 (307), respectively, with an average *N*_p_ of about 179. Between 1998 and 2020, the *N*_p_ trend (Fig. 1, main panel) can be graphically subdivided in three distinct phases (indicated by vertical dashed lines in the main panel of Fig. 1). A Latent phase, between 1998 and 2001, characterized by a slight increase in the number of papers with a minimum and maximum of 1 and 8 papers published in 1998 and 2001 (average *N*_p_ = 4.5). Between 2002 and 2012 a clear Growth phase can be identified, with minimum and maximum number of published papers of 36 and 303, in 2002 and 2012, respectively (average *N*_p_ = 183). This phase presents a significant linear increase (*N*_p_ = 27.027 Year—54 060; *r* = 0.9543; *p* value < 0.001) with an average raise of about 27 papers per year. The Growth phase is followed by a Steady phase between 2013 and 2020, characterized by a minimum and maximum of 222 and 299 papers published in 2018 and 2014, respectively (average *N*_p_ = 260). Normalized citations (*N*_c_) showed a similar trend as suggested by the significant linear relationship between *N*_c_ and *N*_p_ (*N*_c_ = 2.8595 *N*_p_—1.9195; *r* = 0.9727; *p* value < 0.001). Minimum (1.3) and maximum (935) *N*_c_ were reached in 1999 and 2019, respectively (average *N*_c_ = 510).

### Bibliometric mapping

#### Author keywords

**Fig. 2:**
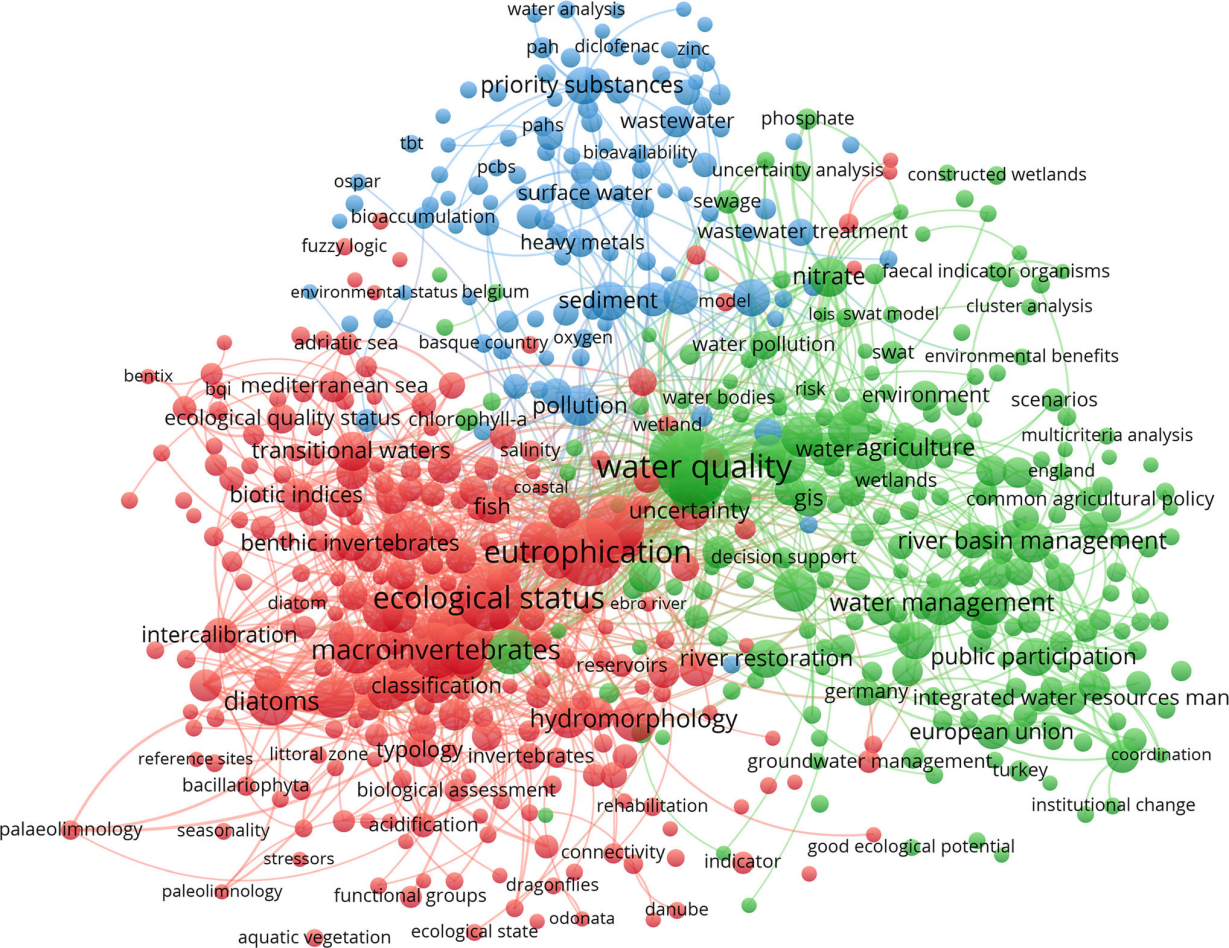
Co-occurrence map of the main 586 author keywords. The map does not contain the keywords: Water Framework Directive and WFD (see “Data and methods” section for details)

A co-occurrence map of author keywords is reported in Fig. 2. The first 50 most frequent keywords used to create this map are listed in Table S3, Water Quality was the one with the highest number of occurrences (253) and links (250). This keyword was the most frequent of the bottom-right (green) cluster 1 and occupied a central position in the co-occurrence network (Fig. 2). Other relevant keywords in the first cluster were: Phosphorus, Nutrients, Water Management, Rivers, Climate Change, Groundwater, Nitrogen, Agriculture, and River Basin Management (Table S3). This cluster seemed oriented to management aspects including the control of nutrient concentrations and climate change (Copetti et al. 2013). Rivers and Groundwater were the most frequent keywords related to waterbodies within this cluster.

The two keywords with the highest occurrences of the bottom-left (red) cluster 2 were Eutrophication (190) and Ecological Status (187), both keywords established a total number of 195 links. Other relevant terms were Monitoring, Macroinvertebrate, Macrophytes, Phytoplankton, Diatoms, Reference Conditions, Hydromorphology, Bioassessment, and Lakes (Table S3). This cluster contained words associated to biological, trophic, and hydro-morphological elements and appeared oriented to the definition of the ecological status (Feio et al. 2021) of surface freshwaters.

The keywords belonging to the top-central (blue) cluster 3 were markedly less frequent than those composing the two previous groups. Pollution (50 occurrences) and Sediment (48), the two most frequent terms, occupied only the 27th and 29th position in the occurrence ranking presented in Table S3. These two keywords established 73 and 88 links, respectively. Other relevant words were Priority Substances, Pesticides, Metals, and Risk Assessment (Table S3). This cluster seemed thus to contain chemical and contamination (Coquery et al. 2005) related terms, while it did not seem to be oriented to a specific class of water bodies.

#### Scientific journals

**Fig. 3:**
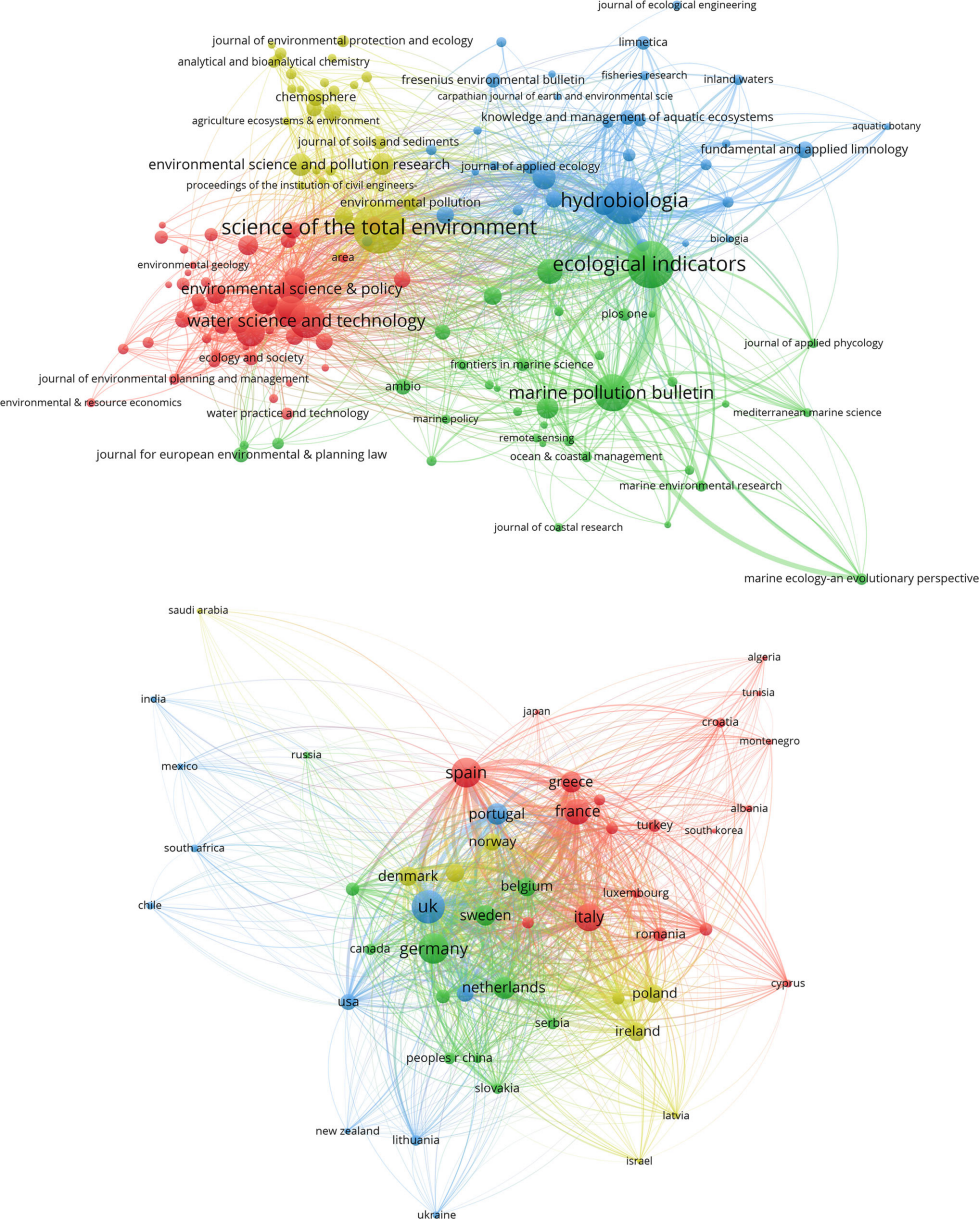
Citation maps of the main 148 scientific journals (upper panel), and 54 countries (lower panel)

The first 50 most productive journals at the basis of the citation map presented in the upper panel of Fig. 3 are listed in Table S4. Among them the ten with the highest number of published articles were: Science of the Total Environment (263), Ecological indicators (225), Hydrobiologia (218), Marine Pollution Bulletin (140), Water Science and Technology (124), Water (93), Water Resources Management (84), Environmental Science & Policy (80), Journal of Environmental Management (67), and Environmental Monitoring and Assessment (65).

Overall, the journals aggregate in four main clusters (Fig. 3, upper panel). The first one on the top-left (yellow) was driven by Science of the Total Environment. This journal established the highest number of links (122) and was associated mainly with chemical and ecotoxicological oriented journals such as Environmental Science and Pollution Research, Water Research, Journal of Environmental Monitoring, Chemosphere, and Environmental Sciences Europe. Moving rightward the second top-right cluster (blue), dominated by Hydrobiologia (100 links), was mainly related to ecological and conservation-oriented journals such as: Limnologica, Aquatic Conservation-Marine and Freshwater Ecosystems, River Research and Applications, and Fundamental and Applied Limnology. The main journal of the bottom-right (green) cluster 3 was Ecological Indicators (98 links). This cluster seemed more related to monitoring issues and focused on marine environments, including journals such as: Marine pollution bulletin, Environmental Monitoring and Assessment, Estuarine Coastal and Shelf Science, and Biology and Environment-Proceedings of the Royal Irish Academy. Moving leftward the fourth cluster was dominated by Water Science and Technology (65 links). This cluster was composed mainly by journals related to environmental technology and water management, such as: Water, Water Resources Management, Environmental Science & Policy, and Journal of Environmental Management.

#### Countries

A citation map of the countries network is reported in the lower panel of Fig. 3 and the 50 more productive countries of the network are listed in Table S5. The first 10 were: UK (855 papers), Germany (658), Spain (623), Italy (502), France (429), Netherlands (274), Portugal (255), Greece (241), Sweden (226), and Denmark (197). UK (50 links), including Scotland, Northern Ireland, and Wales, was the principal country of cluster 1 (blue) which developed mainly on the left part of the network. Lithuania as well as countries from other continents such as: New Zealand, Chile, South Africa, Mexico, and India were also part of this cluster. Below, in a bottom-central position is located cluster 2 (green). The cluster was dominated by Germany (52 links) and other northern EU countries such as: Netherlands, Sweden, and Belgium, plus countries from other continents, such as Russia, Brazil, Canada, and Australia. Moving leftward cluster 3 (red) included Mediterranean and Atlantic countries such as: Spain (53 links), Italy, Greece, Croatia, Montenegro, Portugal, and countries from other continents such as north Africa (e.g., Tunisia, and Algeria), and Asia (e.g., Japan, South Korea). Countries located at the border between Europe and Asia (e.g., Turkey) also belonged to this cluster. Cluster 4 (yellow) is in a top-central position. The main country of this group was Denmark (197 documents; 53 links). The cluster was composed by countries from north-eastern Europe, such as Poland, Finland, Norway, Estonia, Latvia, and others from the middle east (i.e., Israel, Saudi Arabia).

#### Institutions

A citation map of the institutions network is showed in Fig. S4, and the 50 institutions most contributing to paper production are reported in Table S6; among them the first ten were the Environment Agency (106 papers), Aarhus University (82), Swedish University of Agricultural Sciences (75), the Ufz Helmholtz Centre for Environmental Research (68), University of Coimbra (67), Centre for Ecology and Hydrology (67), University of Duisburg-Essen (66), IRSTEA (French National Institute for Research in Science and Technology for the Environment and Agriculture, 60), Hellenic Centre for Marine Research (57), and CSIC (Spanish National Research Council, 55). Institutions aggregate in four main clusters (Fig. S4). In cluster 1 (yellow), on the middle-left part of the network, the main organizations were Environment Agency and Aarhus University. These organizations established the highest number of links (347 and 331, respectively). The cluster also included European Commission, Commission of the European Communities, Finnish Environmental Institute, Norwegian Institute for Water Research, and Leibniz Institute for Freshwater Ecology & Inland Fisheries. The main organization in the upper-central red cluster 2 was the Centre for Ecology and Hydrology (236 links), which was mainly related to the Aristotle university of Thessaloniki, University of Ghent, Delft University of Technology, and the University of Utrecht. The principal institution of the right-central green cluster 3 was the Swedish University of Agricultural Sciences (303 links). Other organizations in the cluster were Ufz Helmholtz Centre for Environmental Research, University of Duisburg-Essen, IRSTEA, and CNR (National Research Council of Italy). The bottom-central blue cluster 4 was dominated by University of Coimbra (220 links). Other relevant institutions were Hellenic Centre for Marine Research, CSIC, University of Lisbon, IFREMER (National Institute for Ocean Science).

### Review analysis

**Fig. 4:**
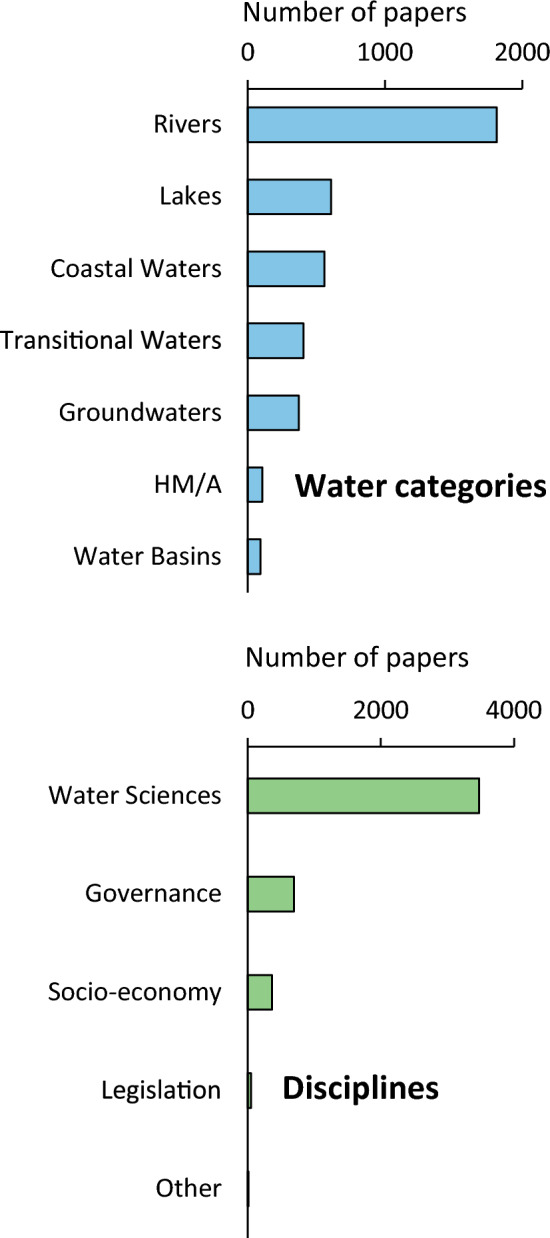
Number of papers dealing with different Water Categories (upper panel) and Disciplines (lower panel) adopted in this study

The most represented water category emerging from the review analysis (Fig. 4, upper panel) was Rivers (1813 papers). This category counted about 3 times more references than Lakes (607) and Coastal Waters (558) and about 4 times more than Transitional Waters (405) and Groundwaters (372).

A comparatively low number of references dealt with Heavily Modified/Artificial (106) and Water Basins (92). Among the different Disciplines (Fig. 4, lower panel), Water Sciences (3473) was the most represented category, followed by Governance (697), and Socio-economy (368). Only a few dozen references referred to other disciplines, including those related to Legislation (51).

**Fig. 5:**
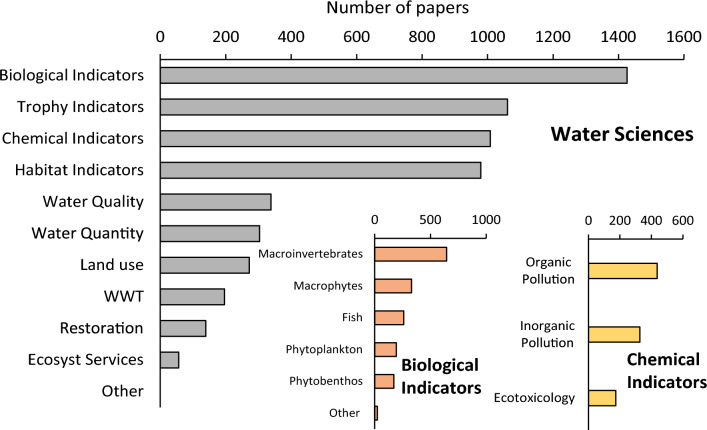
Number of papers dealing with the different Water Sciences (main panel), detail on Biological Indicators (left lower panel), and on Chemical Indicators (right lower panel). WWT stays for Waste Water Treatment

Among Water Sciences (Fig. 5, main panel), most of the references dealt with Biological (1426), Trophy (1061), Chemical (1008), and Habitat (979) Indicators, while the other categories were less represented with number of references between 56 (Ecosystem Services) and 338 (Water Quality).

Most of the references dealing with Biological Indicators (Fig. 5, lower left panel) were related to Macroinvertebrates (646), followed by Macrophytes (330), Fish (261), Phytoplankton (195), and Phytobenthos (172), while references dealing with Other ecological indicators (23) turned out to be negligible. References related to Organic Pollution (436) were the most represented among the articles dealing with chemical aspects (Fig. 5, lower right panel), among the remainder 327 papers faced problems of Inorganic Indicators, and 174 to Ecotoxicology.

**Fig. 6:**
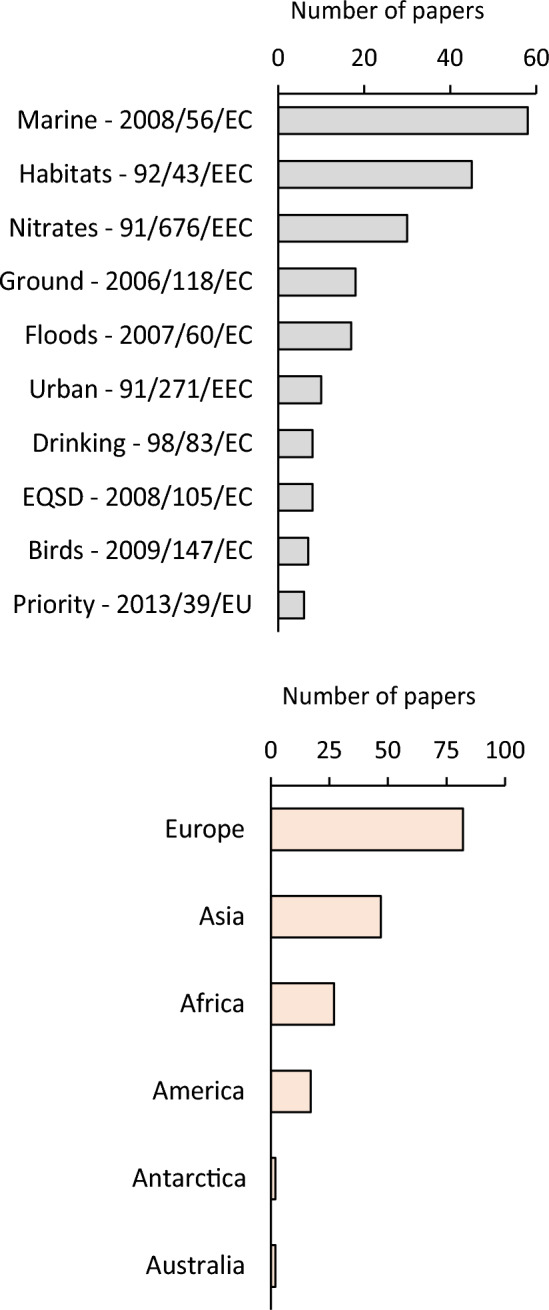
Number of papers citing other EU Directives (upper panel) and extra-EU countries (lower panel)

The number of references directly mentioning other EU Directives was 233. The two most cited Directives (Fig. 6, upper panel) were the Marine Framework Directive-2008/56/EC (58) and the Habitats Directive-92/43/EEC (47). Other Directives with more than 10 citations were: Nitrates Directive-91/676/EEC (31), Groundwater Directive-2006/118/EC (19), and Flood Directive-2007/60/EC (17).

The number of refences reporting experimental activities or applications related to the WFD in extra-EU countries was 177. Most of the papers (Fig. 6, lower panel) dealt with countries located in Europe (82). The other continents with more than 10 references were: Asia (47), Africa (27), and America (17).

## Discussion

### Scientific impact

The results presented in this paper highlight that the WFD has attracted the attention of a multitude of researchers and it has been an important driving force for the scientific community, as indicated by the high number of scientific papers (4120) within the WFD-References Dataset. WFD is one of the most studied pieces of the EU environmental legislation (Boeuf et al. 2016). Even 20 years after its promulgation the production of WFD-related papers is high and almost unvaried since 2012, oscillating around a value of about 260 papers per year. The current steady phase was preceded by a period of high growth in paper production, somehow linked to important and demanding deadlines fixed by the WFD. The year 2009, for instance, corresponded with the definition of the first River Basin Management Plan. This deadline was coupled with the conclusion of the first intercalibration exercise, which forced the scientific community to jointly work on a common approach, to compare the various assessment systems in use in the different member states (e.g., EC 2005; Buffagni et al. 2007; Ruellet and Dauvin 2007; Occhipinti Ambrogi et al. 2009; Erba et al. 2009). Intercalibration results were published even after the conclusion of this first cycle (Poikane et al. 2011), in line with the literature production growth phase (Fig. 1). Other important years identified in the trend of scientific production were 2012 and 2014. This agrees with Boeuf and Fritsch (2016) who argued that, in such years, the highest number of papers was produced, probably in response to the other important deadline of 2015. This deadline has likely stimulated the scientific community to yield consistent scientific bases for the first attempt of reaching the WFD environmental objectives. Moreover, the intercalibration exercise, set out for the Common Implementation Strategy, was still very active during those years, requiring finalization for many assessment systems related to specific water body types (e.g., Poikane et al. 2015) and biological elements (Aguiar et al. 2014; Segurado et al. 2014). Intercalibration per se was a harmonization trial probably never attempted before at such a broad scale and a lot of work was required to develop consistent approaches. Such an exercise entailed a strong collaboration among different countries, resulting in strong paper internationalizations (e.g., Bennett et al., 2011; Erba et al. 2009; Kelly et al. 2009; Poikane et al. 2011; 2015), a further added value related to WFD implementation. The fact that the intercalibration exercise was an important challenge for the scientific community was also emphasized by the results showed in the author keywords map (Fig. 2), in which Intercalibration is among the most important keywords (see also Table S3). The new scientific and management panorama established by the WFD, focusing on ecological aspects, is also well represented by the cluster distribution emerging from this map (Fig. 2). All the three main clusters (focused on water management, ecology, and pollution issues, respectively) present a strict relationship with Water Quality, the most frequent keyword, which occupies a central position in the network. These three macro-groups of keywords represent major subjects of the WFD (Carvalho et al. 2019). Furthermore, the structure of the author keywords network may suggest future directions in the ecological assessment evaluation. Issues represented with smaller circles, for instance, could be associated to topic not much investigated so far and indicating the need for further research. Some of these issues (e.g., metabarcoding, temporary rivers, water scarcity, e-flows, hydro-morphological impact, emerging substances) have been already identified in literature as emerging topics (Moss et al. 2020; Crabot et al. 2021; Blancher et al. 2022) and adopted within the Common Implementation Strategy agenda for 2022–2024. Such results can be used not only in specific studies related to the development of the WFD, but also in a broader research context related to the ecological status assessment. The high number of journals involved (591) is indicative of the heterogeneity (see also Table S3) of the community participating to the scientific development of the WFD. Journals with the largest paper contribution were Science of the Total Environment, Ecological Indicators, and Hydrobiologia. The first identifying a cluster of cross-cutting and multidisciplinary studies, while the other two identifying clusters of ecological studies oriented to marine and freshwater environments, respectively.

### Who has been most involved?

The strength of the scientific collaboration among EU countries clearly emerged from the country citation map (Fig. 5) and represents an indirect success of the WFD implementation. The countries with the highest scientific paper production in the WFD-References Dataset were UK, Germany, Spain, Italy, and France. This partially conflicts with the results presented by Boeuf and Fritsch (2016) who found (analyzing a much smaller samples of 89 papers) that countries in northwestern Europe dominated the scientific production of WFD-linked papers. Apparently, in recent years southern European countries aligned their scientific productivity to that of the northwestern countries. However, a north-south orientation in the clusters of the country citation map is visible: north-east European countries grouped (and inter-linked) together separately from south-west countries, suggesting that the ecoregional approach inherent in the WFD and site-specific issues may have influenced the scientific collaboration among countries. It is not surprising that universities and research centers (e.g., Aarhus University, Swedish University of Agricultural Sciences, Helmholtz Centre for Environmental Research, University of Coimbra CEH, CNR) played a major role in scientific paper production, compared to other non-academic institutions. However, the role of some agencies (e.g., UK Environment Agency) can be as much important as the role of some research institutions. This might be related to the fact that some countries precociously recognized the importance of fully involving practitioners in WFD implementation, boosting their representatives to participate in international contexts, where strategies and directions for the WFD application were taken to optimize the transfer of scientific approaches to applied issues (Kelly et al. 2009; Munné and Prat 2009; Boon et al. 2020). As well, it has to be emphasized that many papers originated from technical reports required from national ministries and European institutions. This enhanced the effort of non-academic organizations and applied agencies to collaborate with research structures to convert such reports in scientific papers. Similar considerations can be advanced for EU institutions (e.g., European Commission).

### Which issues were more explored?

One first relevant result of the review analysis is that rivers are the most studied water bodies. This is probably related to the occurrence and wide distribution of such ecosystems in the EU area, but perhaps also to a greater presence of river ecologists and scientists within the WFD scientific community.

In relation to the different disciplines contributing to the WFD development, it is not surprising that Water Sciences were prominent compared to the other subjects. Various Water Sciences provide fundamental concepts for the protection of aquatic ecosystems, are related to the assessment of the ecological status, and are linked with the main articles of the WFD. However, our results underline that principles from other disciplines introduced by the WFD such as the economic value of ecosystems, the societal inclusion in ecological issues, and the global approach to management and governance issues, received considerable attention from the scientific community (e.g., Deffner and Haase 2018; Sola et al. 2020). Among Water Sciences, studies related to biological communities are the most frequent. This is clearly related to the fact that biological quality elements are the main focus of the WFD (Feio et al. 2021). Moreover, issues related to the trophic evaluation received great attention in the WFD-References Dataset, suggesting that eutrophication-related problems are still tangibles (Bonsdorff 2021; Erba et al. 2022) and evidencing a strict link between trophic issues and more ecologically sound problems (e.g., O’Hare et al. 2018). Another evidence emerging from the review analysis is a confirmation of the relevant role played by macroinvertebrates among the biological elements. This reinforces the long-recognized leading role played by macroinvertebrates in biomonitoring (e.g., Cairns Jr. and Pratt 1993). Among the studies supporting biological element, hydrological, morphological, and chemical approaches received almost the same attention in the dataset.

### Relationships and potential spread

The most frequently cited EU directives in the WFD-References Dataset were Marine, Habitat, and Nitrates Directives. Marine Directive together with WFD, defines the management approach to coastal environments, and it is thus frequently mentioned in marine oriented papers. The attention to the Habitat Directive instead underlines the strict link between the restoration of water environments and the protection of habitats, which should proceed together. Finally, the attention to the Nitrates Directive indicates a high sensitivity of the scientific community to the problem of the diffuse pollution. These insights were partially expected and can be related to the integrated approach defined by the WFD. However, the number of papers simultaneously dealing with WFD and other Directives (233) is globally low, suggesting a scarce sensitivity of the scientific community to the integration between different EU Directives. This result corroborates the findings obtained by Boeuf and Fritsch (2016) on a smaller number of publications.

Most WFD applications in extra-EU countries were related to the European and the Asian continent, while other geographical contexts were less represented. This clearly indicates that many EU neighboring countries (e.g., Norway, Switzerland, Turkey, Serbia, North Macedonia) looked at this Directive to inspire their water protection policies (Fritsch et al. 2020). The reasons behind this interest could also be political, as some of these countries aspire to become EU members. Furthermore, some countries joined EU after 2000 but their willingness to adopt WFD principles started earlier their joining EU, and this may have had a small effect on the results presented in this paper. Overall, these outcomes indicate a spread of the WFD approach outside the EU borders, potentially even in other continents.

## Conclusions, perspectives, and recommendations

After 20 years from its promulgation the scientific production on WFD is still high and remains constantly at values of around 260 papers per year, indicating a lasting attention of the scientific community on this European Directive. In the past two decades such literature production increased in response to major scientific challenges posed by WFD implementation. Most of the studies were related to water sciences, and primarly oriented to aquatic ecology, but consistent contributions were also from governance and socio-economy disciplines. The importance of all these three main approaches emerged from both the bibliometric mapping and the review analysis carried out in this study. Therefore, the WFD scientific production was not monopolized by a single discipline but was, instead, characterized by a moltitude of scientific and methodological approaches, indicating a strong interest in combining environmental and societal needs. Among the papers dealing with aquatic sciences the biological ones were the most represented, followed by chemical, trophy, and habitat-oriented studies. The most frequently cited biological elements were macroinvertebrates, while rivers were the predominant water category in the dataset. The number of references mentioning other EU Directives was overall scarce (about 6%) suggesting a limited interconnection between the WFD and other EU environmetal Directives, at least in academic studies. Clearly, it is hoped that this integration could increase in the next future. As expected, WFD exerted a strong impact on research activities carried out in EU countries, a certain influence is also evident in neighboring nations, and to a lesser extend also in other continents. The close link between scientific production and WFD deadlines emerged in this paper suggests that the attention of the scientific community should remain high at least until the last deadline set for 2027. However, the presence of open issues in the WFD implementation and the advance of new environmental water related problems might deternine a further increase in scientific production. For a more effective management of water resources, policy efforts should be better focused on emerging environmental issues and crossing topics for different European legislations. On this regards the results presented here can be a starting point and help researchers to develop specific research areas within a common integrated vision.

### Supplementary Information

Below is the link to the electronic supplementary material.

Supplementary file1 (PDF 1260 KB)
