# Supplementary material for: A bibliometric review on the Water Framework Directive twenty years after its birth
Source: Ambio. 2023 Sep 8;53(1):95–108. doi: 10.1007/s13280-023-01918-0 (PMC10692058; doi:10.1007/s13280-023-01918-0)
Supplement: Supplementary file 1 — Supplementary file1 (PDF 1260 KB) [file 13280_2023_1918_MOESM1_ESM.pdf]

Ambio

Electronic Supplementary Material
This supplementary information has not been peer reviewed.

Title: **A bibliometric review on the Water Framework Directive twenty years after its birth**

Authors: Diego Copetti, Stefania Erba

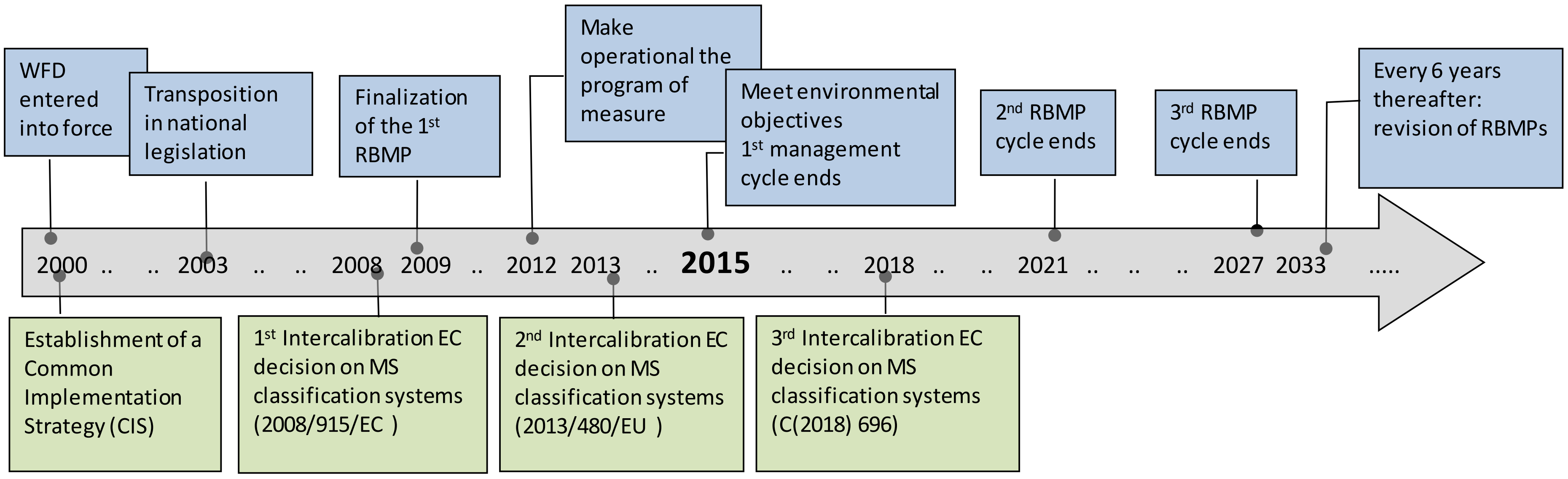

Fig. S1. WFD time schedule. Above the arrow (in blue) is reported the official time table for the WFD implementation; below the arrow (in green) are reported important deadlines linked with the Common Strategy for WFD Implementation (CIS). RBMP: River Basin Management Plan.

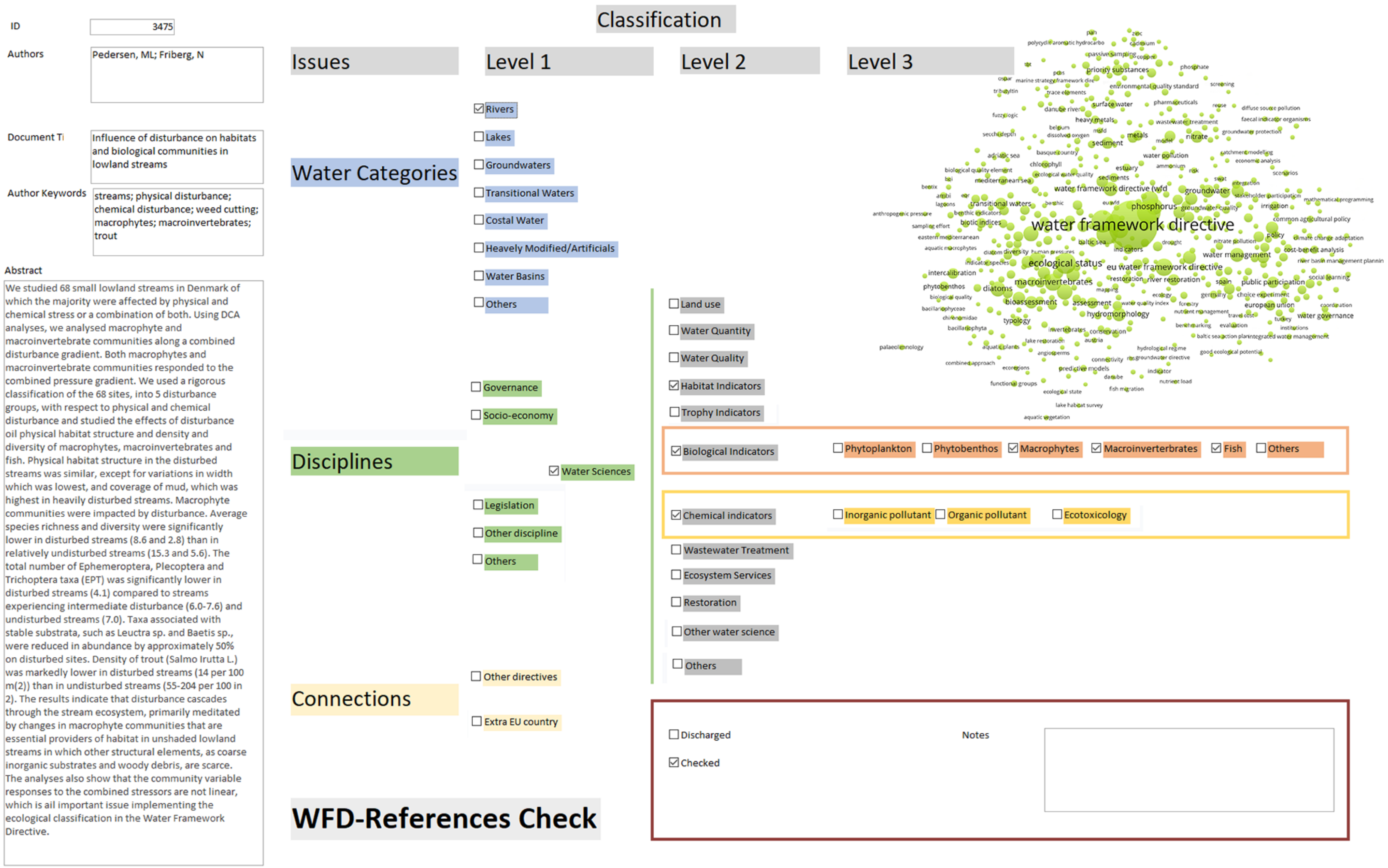

Figure S2. Visual interface (WFD-References Check) developed in Microsoft® Access™ for the analysis of the records imported from the Web of Science core collection database.

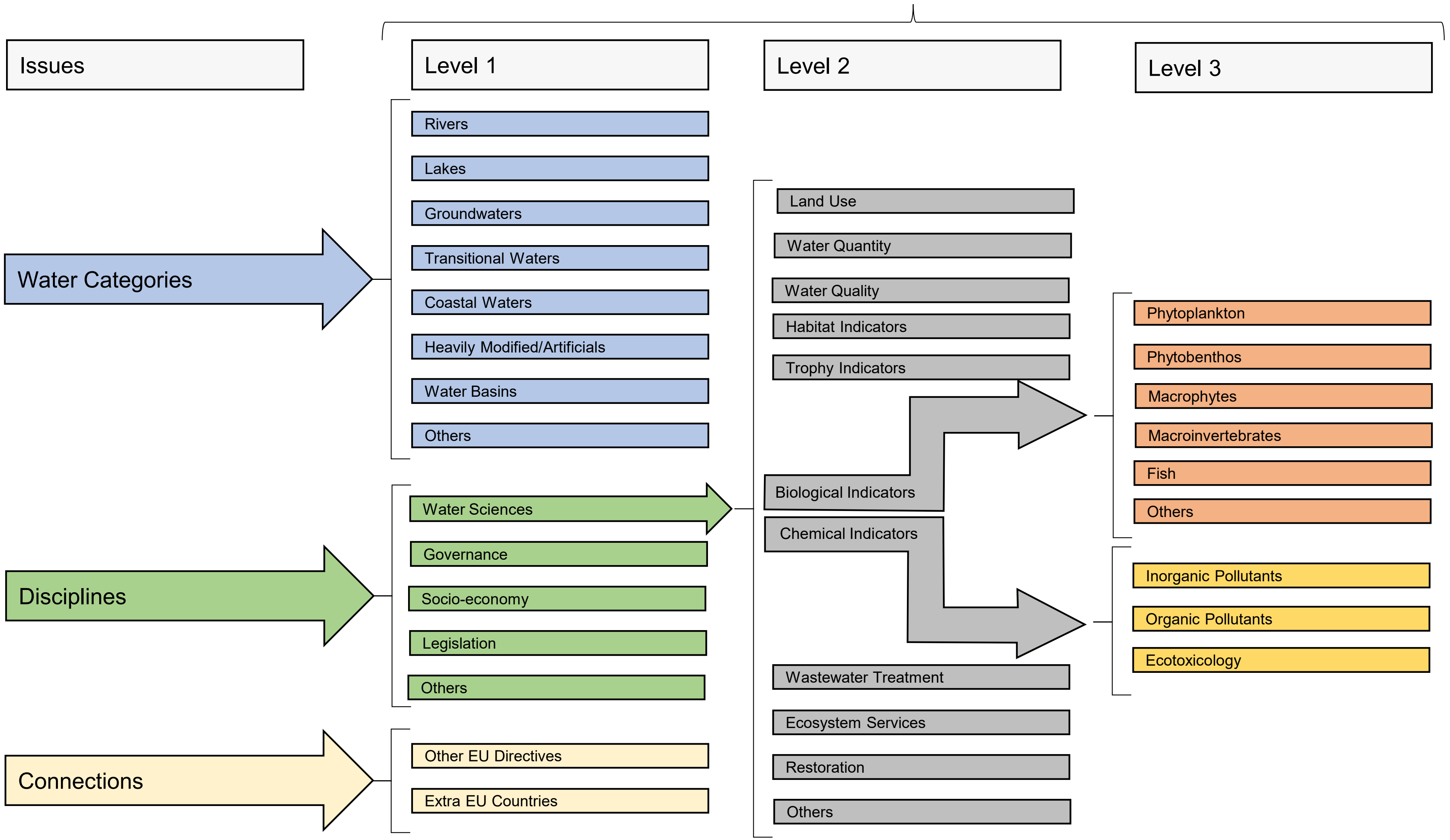

Fig. S3. Classification scheme adopted for the analysis of the papers selected in this study (see text for explanation).

Table S1 Description of the three levels of classification adopted in this study with the list of the items considered and a brief explanation of each item/group. See below the References section for cited articles.

**Level 1**	**Description/Definitions**	**Reference articles**
**Water category**		
Rivers	*Sensu* WFD (i.e., flowing water)	Feio et al., 2014; Rasmussen et al., 2013
Lakes	*Sensu* WFD (i.e., standing water)	Moe et al., 2015
Groundwaters	*Sensu* WFD (i.e., water below the surface of the ground)	Rejman, 2007
Transitional waters	*Sensu* WFD (i.e., surface water bodies in the vicinity of river mouths which are partly saline)	Carstens et al., 2004
Coastal waters	*Sensu* WFD (i.e., water line with a distance of one nautical mile on the seaward side from the nearest point *of the base line)*	Bennet et al., 2011
Heavily Modified/Artificial	*Sensu* WFD (i.e., physically altered water bodies to guarantee a specific water use, indispensable for human life)	Erba et al., 2019
Water Basins	Includes all waterbodies of a catchemt (catchment level approach, without any specification to a particular category)	Medd and Marvin, 2007
Others	Other waterbodies not included in above categories (e.g., wetlands)	Funk et al., 2017
**Discipline**		
Water sciences	Studies directly related to water ecosystems investigation such as water chemistry, biology, ecology, environmental studies etc.	Ramos-Merchante et al., 2021
Governance	Sensu OECD, 2015. "Range of political, institutional and administrative rules, practices and processes (formal and informal) through which decisions are taken and implemented, stakeholders can articulate their interests and have their concerns considered, and decision makers are held accountable for water management". Includes: WFD implementation, decision making, water policy, cost-effectiveness etc.	Valinia et al., 2012
Socio-economy	Socio-economic studies focused on topic such as: water pricing, natural capital, willingness to pay etc.	Hynes and O'Donoghue, 2020
Legislation	Political and legal studies, including presence of legislative incongruency in the application of the WFD	Grönlund and Määttä, 2008
Others	Used for papers dealing with residual approaches not dealing with the above mentioned	Dalbeck and Weinberg, 2009
**Connection**		
Other EU Directives	Explicit reference to other European Directives such as: Habitat Directive, Groundwater Directive etc.	Weigelhofer et al., 2020
Extra EU Countries	Explicitely referring to extra EU countries	Langhans et al., 2015
**Level 2**		
**Water science**		
Land Use	One of the main anthoropogenic pressures that have to be characterized according to WFD. Includes land management (e.g., papers oriented on the management riparian areas and of diffusive nutrient loads from agricultural areas)	Krause et al., 2008
Water Quantity	Generic hydrological issues not directly considered as a WFD supporting element (e.g., when water is used for drinking or agricultural supply or strictly hydrological oriented papers)	Bizzi et al., 2012
Water Quality	Generic water quality issues, not classifiable using issues Biological, Trophi, or Chemical Indicators. These papers often refer to rather generic approaches such as water quality management or similar	Freni et al., 2009
Habitat Indicators	Habitat here considered in a broad sense, including all possible habitat-related features supporting biological elements sensu WFD, such as hydro-morphological conditions - morphological and hydrological - supporting elements, chemical and physico-chemical supporting elements - excluding nutrients and oxygen - supporting the biological elements, general supporting elements	Mueller et al., 2011
Trophy indicators	Variables supporting the ecological status classification directly or undirectly related to nutrient conditions (e.g., nitrogen, phosporous, and oxygen concentrations or others like chlorophylla-a). When used as indicator of trophic state chlorophyll-a was considered a Trophy indicator while when used as a proxy of the phytoplankton population it was considered as a Biology indicator, this happen frequently in papers related to costal or transitional areas and in studies dealing with remote sensing. For homogeneity with other water bodies nitrate was considered a Trophy indicator even in ground water environments, even if for this water bodies it should be considered as an indicator of chemical status	Andersen et al., 2016
Biological Indicators	Biological quality elements *sensu* WFD	Sigamani et al., 2015
Chemical Indicators	Chemical quality elements, contributing to the definition of chemical status *sensu* WFD	Gevrey et al., 2010
Wastewater Treatment	Water quality issues directly linked to treatment or processed wastewater often considered as a prerequisite to meet the aims of the WFD	Corominas et al., 2013
Ecosystem Services	The concept of ecosystem service as defined by MA [Millennium Ecosystem Assessment] 2005 (http://www.millenniumassessment.org/en/About.html): "the benefits people derive from ecosystems" and by Fisher and Turner, 2008 "the aspects of ecosystems utilized (actively or passively) to produce human well-being".	Rova et al., 2015
Restoration	The imporvement of the ecological status and quality trought the application of passive or active measures (e.g., geo-engineering case studies, biomanipulation approaches)	Kristensen et al., 2011
Others	All the residual papers not dealing with the above described issues.	Moss, 2008
**Level 3**		
**Biological indicator**		
Phytoplankton	*sensu* WFD (apply to all waters except rivers and groundwaters)	Couture et al., 2018
Phytobenthos	*sensu* WFD (apply to all waters except groundwaters)	Tison et al., 2008
Macrophytes	*sensu* WFD (all waters except groundwaters)	Leyssen et al., 2014
Macroinvertebrates	*sensu* WFD (all waters except groundwaters)	Erba et al., 2015; Kail et al. 2012
Fish	*sensu* WFD (all waters except groundwaters)	Wright et al., 2017
Others	e.g., bacteria, zooplankton (all waters)	Carvalho et al., 2008
**Chemical indicator**		
Inorganic Pollutants	Metals, elements and their compounds (e.g., arsenic and its compounds)	Paraskevopoulou et al., 2014
Organic Pollutants	Organic compounds, including pharmaceuticals and biocides	Hutchinson et al., 2013
Ecotoxicology	Study of the toxic effects of chemical and physical agents on all living organisms (using test organisms)	Palma et al., 2016

Table S2. List of first research areas used by Web of Science to classify the 4120 references in the WFD-References Dataset and respective number of papers. Horizontal lines are placed every ten research areas.

First research area	Papers
Environmental Sciences & Ecology	1725
Engineering	503
Marine & Freshwater Biology	482
Biodiversity & Conservation	273
Water Resources	176
Chemistry	142
Geology	103
Fisheries	91
Agriculture	78
Science & Technology - Other Topics	71
Life Sciences & Biomedicine - Other Topics	50
Biochemistry & Molecular Biology	45
Business & Economics	43
Computer Science	40
Plant Sciences	38
Government & Law	37
Geography	35
Oceanography	29
Physical Geography	25
Zoology	25
Biotechnology & Applied Microbiology	21
Development Studies	17
Geochemistry & Geophysics	11
Meteorology & Atmospheric Sciences	5
Public Administration	5
Remote Sensing	5
Social Sciences - Other Topics	5
Education & Educational Research	3
Forestry	3
Metallurgy & Metallurgical Engineering	3
Operations Research & Management Science	3
Public, Environmental & Occupational Health	3
Thermodynamics	3
Toxicology	3
Acoustics	2
Biophysics	2
Energy & Fuels	2
International Relations	2
Arts & Humanities - Other Topics	1
Electrochemistry	1
Endocrinology & Metabolism	1
Entomology	1
Food Science & Technology	1
Materials Science	1
Mathematical Methods In Social Sciences	1
Paleontology	1
Sociology	1
Transportation	1
Urban Studies	1

Table S3. Fifty most frequent author keywords used to outline Fig. 2 and indication of the respective cluster number, occurrences, and number of direct links with other keywords. Data are sorted based on the number of occurrences. Horizontal lines are placed every ten keywords.

Author keyword	Cluster	Occurrences	Direct links
water quality	1	253	250
eutrophication	2	190	195
ecological status	2	187	195
monitoring	2	134	168
macroinvertebrates	2	118	141
phosphorus	1	104	105
nutrients	1	103	140
macrophytes	2	93	113
phytoplankton	2	88	110
diatoms	2	83	89
reference conditions	2	78	93
water management	1	70	105
rivers	1	68	97
hydromorphology	2	66	91
climate change	1	62	86
groundwater	1	60	85
bioassessment	2	56	73
lakes	2	56	78
nitrogen	1	56	82
ecological quality	2	55	84
agriculture	1	55	92
river basin management	1	55	78
biomonitoring	2	53	79
modelling	1	53	75
nitrate	1	52	64
water policy	1	50	66
pollution	3	50	88
transitional waters	2	49	70
sediment	3	48	73
uncertainty	2	47	59
river	2	46	88
public participation	1	46	66
river restoration	1	45	68
priority substances	3	44	48
assessment	2	43	84
water resources	1	43	51
pesticides	3	43	70
gis	1	42	73
intercalibration	2	39	56
ecosystem services	1	39	63
diffuse pollution	1	38	59
lake	2	36	53
european union	1	36	59
land use	1	36	62
metals	3	36	52
classification	2	35	58
benthic invertebrates	2	34	60
biotic indices	2	34	55
europe	2	34	62
macroalgae	2	34	43

Table S4. Fifty most productive journals used to outline the upper panel of Fig. 3 and indication of the respective cluster number, published papers (Documents), number of direct links with other journals, citations, average publication year (APY), and average citations (AC). Data are sorted based on the number of published articles. Horizontal lines are placed every ten journals.

Journal	Cluster	Documents	Direct links	Citations	APY	AC
science of the total environment	1	263	122	9439	2015	35.9
ecological indicators	3	225	98	6384	2014	28.4
hydrobiologia	2	218	100	8406	2010	38.6
marine pollution bulletin	3	140	81	6951	2011	49.7
water science and technology	4	124	65	1305	2008	10.5
water	4	93	84	700	2018	7.5
water resources management	4	84	72	1879	2012	22.4
environmental science & policy	4	80	98	2861	2011	35.8
journal of environmental management	4	67	86	1923	2012	28.7
environmental monitoring and assessment	3	65	77	1168	2012	18.0
limnologica	2	56	64	1478	2010	26.4
aquatic conservation-marine and freshwater ecosystems	2	54	78	2430	2008	45.0
environmental science and pollution research	1	52	51	871	2014	16.8
water research	1	49	54	2010	2013	41.0
estuarine coastal and shelf science	3	48	55	2033	2012	42.4
environmental earth sciences	4	41	42	415	2015	10.1
journal of hydrology	4	40	55	1932	2010	48.3
water and environment journal	4	40	51	592	2009	14.8
water policy	4	36	51	395	2013	11.0
journal of environmental monitoring	1	34	55	720	2008	21.2
biology and environment-proceedings of the royal irish academy	3	33	40	255	2012	7.7
river research and applications	2	31	46	872	2014	28.1
environmental modelling & software	4	30	48	2052	2010	68.4
chemosphere	1	29	27	918	2012	31.7
hydrology and earth system sciences	4	29	31	927	2009	32.0
land use policy	4	29	56	1324	2013	45.7
environmental sciences europe	1	28	27	520	2019	18.6
fundamental and applied limnology	2	28	26	395	2012	14.1
international journal of river basin management	4	28	49	254	2011	9.1
environmental management	4	27	72	576	2012	21.3
freshwater biology	2	27	59	2216	2009	82.1
environmental pollution	1	26	32	803	2012	30.9
ambio	3	25	42	461	2012	18.4
physics and chemistry of the earth	4	24	44	599	2007	25.0
ecological economics	4	23	42	1289	2011	56.0
ecological modelling	3	23	43	1232	2009	53.6
journal for european environmental & planning law	3	23	18	64	2013	2.8
trac-trends in analytical chemistry	1	23	34	731	2009	31.8
environmental policy and governance	4	22	30	402	2015	18.3
fresenius environmental bulletin	2	22	17	94	2011	4.3
knowledge and management of aquatic ecosystems	2	22	35	155	2015	7.0
ecological engineering	4	21	38	505	2012	24.0
water international	4	20	29	159	2011	8.0
ecology and society	4	19	41	976	2011	51.4
journal of applied ecology	2	19	43	1456	2010	76.6
journal of soils and sediments	1	19	17	423	2012	22.3
limnetica	2	19	21	180	2014	9.5
desalination	4	18	24	548	2008	30.4
desalination and water treatment	4	18	16	121	2015	6.7
international journal of water resources development	4	18	25	238	2009	13.2

Table S5. Fifty most productive countries used to outline the lower panel of Fig. 3 and indication of the respective cluster number, published papers (Documents), number of direct links with other countries, citations, average publication year (APY), and average citations (AC). Data are sorted based on the number of published articles. Horizontal lines are placed every ten countries.

Country	Cluster	Documents	Direct links	Citations	APY	AC
england	1	693	53	25024	2011	36.1
germany	2	658	52	19860	2012	30.2
spain	3	623	53	19968	2013	32.1
italy	3	502	52	13952	2013	27.8
france	3	429	53	14679	2013	34.2
netherlands	2	274	50	9827	2012	35.9
portugal	3	255	53	7359	2013	28.9
greece	3	241	52	6366	2013	26.4
sweden	2	226	51	8142	2013	36.0
scotland	1	219	50	7137	2011	32.6
denmark	4	197	53	7755	2012	39.4
belgium	2	183	51	6442	2012	35.2
poland	4	169	49	3459	2014	20.5
ireland	1	165	48	4166	2013	25.2
finland	4	156	49	5542	2012	35.5
norway	4	142	51	5221	2013	36.8
austria	2	123	49	5059	2011	41.1
usa	2	115	48	5066	2012	44.1
czech republic	2	75	49	2579	2014	34.4
romania	3	75	45	1553	2014	20.7
switzerland	2	68	43	3081	2014	45.3
hungary	3	61	42	1175	2014	19.3
turkey	3	59	44	925	2016	15.7
wales	1	57	42	1895	2010	33.2
estonia	4	55	45	2060	2012	37.5
north ireland	1	49	44	1814	2011	37.0
australia	2	49	43	2351	2013	48.0
slovenia	3	48	48	1137	2014	23.7
peoples r china	2	45	38	1207	2015	26.8
serbia	2	41	38	718	2016	17.5
canada	2	39	39	1557	2014	39.9
bulgaria	3	39	43	626	2012	16.1
slovakia	2	38	37	1371	2013	36.1
lithuania	1	36	39	686	2014	19.1
croatia	3	32	35	479	2016	15.0
luxembourg	3	23	40	610	2011	26.5
brazil	2	20	36	746	2017	37.3
cyprus	3	17	33	529	2014	31.1
chile	1	16	20	144	2016	9.0
south africa	1	15	27	345	2014	23.0
ukraine	1	13	26	306	2017	23.5
albania	3	13	32	107	2017	8.2
mexico	1	11	25	146	2015	13.3
russia	2	11	19	197	2014	17.9
latvia	4	9	37	295	2012	32.8
india	1	9	19	72	2015	8.0
new zealand	1	9	28	356	2010	39.6
algeria	3	7	22	152	2013	21.7
montenegro	3	6	31	57	2019	9.5
tunisia	3	6	19	224	2011	37.3

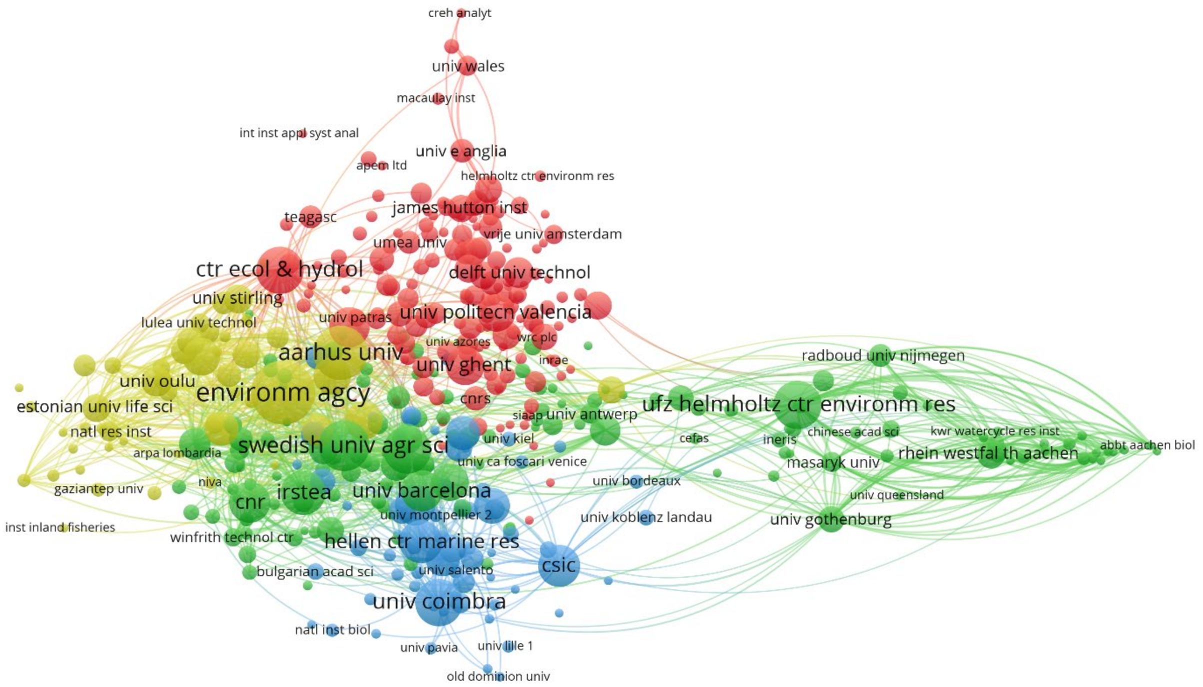

Fig. S4 Citation maps of the main 349 institutions

Table S6. Fifty most productive institutions used to outline Fig. S4 and indication of the respective cluster number, published papers (Documents), number of direct links with other institutions, citations, average publication year (APY), and average citations (AC). Data are sorted based on the number of published articles. Horizontal lines are placed every ten institutions.

Institution	Cluster	Documents	Direct links	Citations	APY	AC
environm agcy	1	106	347	4021	2011	37.9
aarhus univ	1	82	331	3329	2014	40.6
swedish univ agr sci	3	75	303	2762	2013	36.8
ufz helmholtz ctr environm res	3	68	248	3003	2013	44.2
univ coimbra	4	67	220	1872	2012	27.9
ctr ecol & hydrol	2	67	236	2691	2010	40.2
univ duisburg essen	3	66	327	3632	2014	55.0
irstea	3	60	263	2018	2014	33.6
hellen ctr marine res	4	57	233	1211	2014	21.2
csic	4	55	240	2046	2013	37.2
cnr	3	54	251	1783	2012	33.0
univ barcelona	3	54	231	1716	2012	31.8
aristotle univ thessaloniki	2	52	179	719	2014	13.8
univ lisbon	4	51	237	1418	2014	27.8
univ ghent	2	48	220	2229	2011	46.4
european commiss	1	47	305	1886	2015	40.1
commiss european communities	1	47	292	2207	2009	47.0
univ aveiro	3	47	239	946	2014	20.1
finnish environm inst	1	46	171	1057	2011	23.0
univ politecn valencia	2	46	151	1060	2014	23.0
ifremer	4	43	176	1708	2014	39.7
norwegian inst water res	1	40	235	1331	2012	33.3
azti tecnalia	4	40	272	3416	2011	85.4
univ murcia	4	39	191	1434	2012	36.8
leibniz inst freshwater ecol & inland fisheries	1	36	198	1140	2011	31.7
bowburn consultancy	3	35	230	1338	2014	38.2
univ oulu	1	34	173	1647	2012	48.4
norwegian inst water res niva	3	33	244	1680	2014	50.9
univ porto	3	33	172	421	2014	12.8
delft univ technol	2	33	82	414	2012	12.5
univ girona	3	32	208	1233	2011	38.5
univ stirling	1	31	208	1475	2012	47.6
inra	3	30	171	1191	2013	39.7
estonian univ life sci	1	29	185	1142	2014	39.4
environm protect agcy	1	29	182	703	2015	24.2
rhein westfal th aachen	3	29	91	908	2016	31.3
univ utrecht	2	29	157	1358	2014	46.8
univ complutense madrid	2	29	132	929	2012	32.0
cranfield univ	2	29	109	586	2012	20.2
finnish environm inst syke	1	28	242	1141	2014	40.8
deltares	1	28	189	854	2014	30.5
univ algarve	4	28	136	817	2012	29.2
univ aegean	4	28	104	836	2012	29.9
univ vigo	3	28	200	757	2014	27.0
james hutton inst	2	28	170	759	2015	27.1
univ leeds	2	28	162	949	2012	33.9
univ copenhagen	2	28	157	757	2014	27.0
univ valencia	2	28	91	729	2015	26.0
tech univ denmark	2	27	97	1151	2013	42.6
univ hull	4	27	170	1773	2011	65.7

References

Andersen, J.H., Aroviita, J., Carstensen, J., Friberg, N., Johnson, R.K., Kauppila, P., Lindegarth, M., Murray, C., Norling, K., 2016. Approaches for integrated assessment of ecological and eutrophication status of surface waters in Nordic Countries. Ambio 45, 681–691. https://doi.org/10.1007/s13280-016-0767-8

Bennett, S., Roca, G., Romero, J., Alcoverro, T., 2011. Ecological status of seagrass ecosystems: An uncertainty analysis of the meadow classification based on the Posidonia oceanica multivariate index (POMI). Mar. Pollut. Bull. 62, 1616–1621. https://doi.org/10.1016/j.marpolbul.2011.06.016

Bizzi, S., Pianosi, F., Soncini-Sessa, R., 2012. Valuing hydrological alteration in multi-objective water resources management. J. Hydrol. 472–473, 277–286. https://doi.org/10.1016/j.jhydrol.2012.09.033

Carstens, M., Claussen, U., Bergemann, M., Gaumert, T., 2004. Transitional waters in Germany: the Elbe estuary as an example. Aquat. Conserv. Mar. Freshw. Ecosyst. 14, S81–S92. https://doi.org/10.1002/aqc.652

Carvalho, L., Solimini, A., Phillips, G., van den Berg, M., Pietiläinen, O.-P., Lyche Solheim, A., Poikane, S., Mischke, U., 2008. Chlorophyll reference conditions for European lake types used for intercalibration of ecological status. Aquat. Ecol. 42, 203–211. https://doi.org/10.1007/s10452-008-9189-4

Corominas, L., Acuña, V., Ginebreda, A., Poch, M., 2013. Integration of freshwater environmental policies and wastewater treatment plant management. Sci. Total Environ. 445–446, 185–191. https://doi.org/10.1016/j.scitotenv.2012.12.055

Couture, R.-M., Moe, S.J., Lin, Y., Kaste, Ø., Haande, S., Lyche Solheim, A., 2018. Simulating water quality and ecological status of Lake Vansjø, Norway, under land-use and climate change by linking process-oriented models with a Bayesian network. Sci. Total Environ. 621, 713–724. https://doi.org/10.1016/j.scitotenv.2017.11.303

Dalbeck, L., Weinberg, K., 2009. Artificial ponds: a substitute for natural Beaver ponds in a Central European Highland (Eifel, Germany)? Hydrobiologia 630, 49–62. https://doi.org/10.1007/s10750-009-9779-8

Erba, S., Pace, G., Demartini, D., Di Pasquale, D., Dörflinger, G., Buffagni, A., 2015. Land use at the reach scale as a major determinant for benthic invertebrate community in Mediterranean rivers of Cyprus. Ecol. Indic. 48, 477–491. https://doi.org/10.1016/j.ecolind.2014.09.010

Erba, S., Terranova, L., Cazzola, M., Cason, M., Buffagni, A., 2019. Defining Maximum Ecological Potential for heavily modified lowland streams of Northern Italy. Sci. Total Environ. 684, 196–206. https://doi.org/10.1016/j.scitotenv.2019.05.348

Feio, M.J., Aguiar, F.C., Almeida, S.F.P., Ferreira, J., Ferreira, M.T., Elias, C., Serra, S.R.Q., Buffagni, A., Cambra, J., Chauvin, C., Delmas, F., Dörflinger, G., Erba, S., Flor, N., Ferréol, M., Germ, M., Mancini, L., Manolaki, P., Marcheggiani, S., Minciardi, M.R., Munné, A., Papastergiadou, E., Prat, N., Puccinelli, C., Rosebery, J., Sabater, S., Ciadamidaro, S., Tornés, E., Tziortzis, I., Urbanič, G., Vieira, C., 2014. Least Disturbed Condition for European Mediterranean rivers. Sci. Total Environ. 476–477, 745–756. https://doi.org/10.1016/j.scitotenv.2013.05.056

Freni, G., Mannina, G., Viviani, G., 2009. Uncertainty assessment of an integrated urban drainage model. J. Hydrol. 373, 392–404. https://doi.org/10.1016/j.jhydrol.2009.04.037

Funk, A., Trauner, D., Reckendorfer, W., Hein, T., 2017. The Benthic Invertebrates Floodplain Index – Extending the assessment approach. Ecol. Indic. 79, 303–309. https://doi.org/10.1016/j.ecolind.2017.04.035

Gevrey, M., Comte, L., de Zwart, D., de Deckere, E., Lek, S., 2010. Modeling the chemical and toxic water status of the Scheldt basin (Belgium), using aquatic invertebrate assemblages and an advanced modeling method. Environ. Pollut. 158, 3209–3218. https://doi.org/10.1016/j.envpol.2010.07.006

Grönlund, E., Määttä, T., 2008. Implications of flexibility in European Community environmental law: exemptions from environmental objectives in the Water Framework Directive. Hydrobiologia 599, 221–226. https://doi.org/10.1007/s10750-007-9214-y

Hutchinson, T.H., Lyons, B.P., Thain, J.E., Law, R.J., 2013. Evaluating legacy contaminants and emerging chemicals in marine environments using adverse outcome pathways and biological effects-directed analysis. Mar. Pollut. Bull., The Global State of the Ocean; Interactions Between Stresses, Impacts and Some Potential Solutions. Synthesis papers from the International Programme on the State of the Ocean 2011 and 2012 Workshops 74, 517–525. https://doi.org/10.1016/j.marpolbul.2013.06.012

Hynes, S., O'Donoghue, C., 2020. Value transfer using spatial microsimulation modelling: Estimating the value of achieving good ecological status under the EU Water Framework Directive across catchments. Environ. Sci. Policy 110, 60–70. https://doi.org/10.1016/j.envsci.2020.05.006

Kail, J., Arle, J., Jähnig, S.C., 2012. Limiting factors and thresholds for macroinvertebrate assemblages in European rivers: Empirical evidence from three datasets on water quality, catchment urbanization, and river restoration. Ecol. Indic. 18, 63–72. https://doi.org/10.1016/j.ecolind.2011.09.038

Krause, S., Jacobs, J., Voss, A., Bronstert, A., Zehe, E., 2008. Assessing the impact of changes in landuse and management practices on the diffuse pollution and retention of nitrate in a riparian floodplain. Sci. Total Environ. 389, 149–164. https://doi.org/10.1016/j.scitotenv.2007.08.057

Kristensen, E.A., Baattrup-Pedersen, A., Thodsen, H., 2011. An evaluation of restoration practises in lowland streams: Has the physical integrity been re-created? Ecol. Eng. 37, 1654–1660. https://doi.org/10.1016/j.ecoleng.2011.06.043

Langhans, S.D., Reichert, P., Schuwirth, N., 2014. The method matters: A guide for indicator aggregation in ecological assessments. Ecol. Indic. 45, 494–507. https://doi.org/10.1016/j.ecolind.2014.05.014

Leyssen, A., Denys, L., Schneiders, A., Mouton, A.M., 2014. Distribution and environmental requirements of stream habitat with Ranunculion fluitantis and Callitricho-Batrachion vegetation in lower Belgium (Flanders). Aquat. Conserv. Mar. Freshw. Ecosyst. 24, 601–622. https://doi.org/10.1002/aqc.2438

Medd, W., Marvin, S., 2007. Strategic intermediation: between regional strategy and local practice. Sustain. Dev. 15, 318–327.

Moe, S.J., Lyche Solheim, A., Soszka, H., Gołub, M., Hutorowicz, A., Kolada, A., Picińska-Fałtynowicz, J., Białokoz, W., 2015. Integrated assessment of ecological status and misclassification of lakes: The role of uncertainty and index combination rules. Ecol. Indic. 48, 605–615. https://doi.org/10.1016/j.ecolind.2014.08.018

Moss, B., 2008. The Water Framework Directive: Total environment or political compromise? Sci. Total Environ. 400, 32–41. https://doi.org/10.1016/j.scitotenv.2008.04.029

Mueller, M., Pander, J., Geist, J., 2011. The effects of weirs on structural stream habitat and biological communities. J. Appl. Ecol. 48, 1450–1461. https://doi.org/10.1111/j.1365-2664.2011.02035.x

Palma, P., Ledo, L., Alvarenga, P., 2016. Ecotoxicological endpoints, are they useful tools to support ecological status assessment in strongly modified water bodies? Sci. Total Environ. 541, 119–129. https://doi.org/10.1016/j.scitotenv.2015.09.014

Paraskevopoulou, V., Zeri, C., Kaberi, H., Chalkiadaki, O., Krasakopoulou, E., Dassenakis, M., Scoullos, M., 2014. Trace metal variability, background levels and pollution status assessment in line with the water framework and Marine Strategy Framework EU Directives in the waters of a heavily impacted Mediterranean Gulf. Mar. Pollut. Bull. 87, 323–337. https://doi.org/10.1016/j.marpolbul.2014.07.054

Ramos-Merchante, A., Sáez-Gómez, P., Prenda, J., 2021. Historical distribution of freshwater fishes and the reference conditions concept in a large Mediterranean basin. Aquat. Conserv. Mar. Freshw. Ecosyst. 31, 888–902. https://doi.org/10.1002/aqc.3521

Rasmussen, J.J., McKnight, U.S., Loinaz, M.C., Thomsen, N.I., Olsson, M.E., Bjerg, P.L., Binning, P.J., Kronvang, B., 2013. A catchment scale evaluation of multiple stressor effects in headwater streams. Sci. Total Environ. 442, 420–431. https://doi.org/10.1016/j.scitotenv.2012.10.076

Rejman, W., 2007. EU Water Framework Directive versus Real Needs of Groundwater Management. Water Resour. Manag. 21, 1363–1372. https://doi.org/10.1007/s11269-006-9088-1

Rova, S., Pranovi, F., Müller, F., 2015. Provision of ecosystem services in the lagoon of Venice (Italy): an initial spatial assessment. Ecohydrol. Hydrobiol. 15, 13–25. https://doi.org/10.1016/j.ecohyd.2014.12.001

Sigamani, S., Perumal, M., Arumugam, S., Preetha Mini Jose, H.M., Veeraiyan, B., 2015. AMBI indices and multivariate approach to assess the ecological health of Vellar–Coleroon estuarine system undergoing various human activities. Mar. Pollut. Bull. 100, 334–343. https://doi.org/10.1016/j.marpolbul.2015.08.028

Tison, J., Giraudel, J.-L., Coste, M., 2008. Evaluating the ecological status of rivers using an index of ecological distance: An application to diatom communities. Ecol. Indic. 8, 285–291. https://doi.org/10.1016/j.ecolind.2007.02.006

Valinia, S., Hansen, H.-P., Futter, M.N., Bishop, K., Sriskandarajah, N., Fölster, J., 2012. Problems with the reconciliation of good ecological status and public participation in the Water Framework Directive. Sci. Total Environ. 433, 482–490. https://doi.org/10.1016/j.scitotenv.2012.06.087

Weigelhofer, G., Feldbacher, E., Trauner, D., Pölz, E., Hein, T., Funk, A., 2020. Integrating Conflicting Goals of the EC Water Framework Directive and the EC Habitats Directives Into Floodplain Restoration Schemes. Front. Environ. Sci. 8.

Wright, R.F., Couture, R.-M., Christiansen, A.B., Guerrero, J.-L., Kaste, Ø., Barlaup, B.T., 2017. Effects of multiple stresses hydropower, acid deposition and climate change on water chemistry and salmon populations in the River Otra, Norway. Sci. Total Environ. 574, 128–138. https://doi.org/10.1016/j.scitotenv.2016.09.044
